# Issues of under-representation in quantitative DNA metabarcoding weaken the inference about diet of the tundra vole *Microtus oeconomus*

**DOI:** 10.7717/peerj.11936

**Published:** 2021-08-26

**Authors:** Magne Neby, Stefaniya Kamenova, Olivier Devineau, Rolf A. Ims, Eeva M. Soininen

**Affiliations:** 1 Department of Applied Ecology, Inland Norway University of Applied Sciences, Koppang, Norway; 2 Department of Biosciences, University of Oslo, Oslo, Norway; 3 Department of Arctic and Marine Biology, UiT—the Arctic University of Norway, Tromsø, Norway

**Keywords:** DNA diet analysis, High-throughput sequencing, Feeding trial, Rodent, Herbivore, Dietary metabarcoding, Food proportions

## Abstract

During the last decade, methods based on high-throughput sequencing such as DNA metabarcoding have opened up for a range of new questions in animal dietary studies. One of the major advantages of dietary metabarcoding resides in the potential to infer a quantitative relationship between sequence read proportions and biomass of ingested food. However, this relationship’s robustness is highly dependent on the system under study, calling for case-specific assessments. Herbivorous small rodents often play important roles in the ecosystem, and the use of DNA metabarcoding for analyses of rodent diets is increasing. However, there has been no direct validation of the quantitative reliability of DNA metabarcoding for small rodents. Therefore, we used an experimental approach to assess the relationship between input plant biomass and sequence reads proportions from DNA metabarcoding in the tundra vole *Microtus oeconomus.* We found a weakly positive relationship between the number of high-throughput DNA sequences and the expected biomass proportions of food plants. The weak relationship was possibly caused by a systematic under-amplification of one of the three plant taxa fed. Generally, our results add to the growing evidence that case-specific validation studies are required to reliably make use of sequence read abundance as a proxy of relative food proportions in the diet.

## Introduction

Knowledge about animals’ fundamental needs, such as food choice, is central in ecology. Knowing how many different food taxa a species consumes, what these food taxa are, and their quantitative contribution to the overall diet are important questions. Indeed, overall diet composition and the relative contribution of food items with different nutritional content have repercussions to individuals health and growth (Boutin, 1990), which in turn affect population dynamics (Huitu et al., 2007), food web dynamics (Ims et al., 2013), and ecosystem functioning (Schaus, Vanni & Wissing, 2002). Increased knowledge about diet can improve our understanding of the ecological and conservation needs of a particular species (Balmford, Green & Murray, 1996; Bohmann et al., 2014; Elfström et al., 2014; Kowalczyk et al., 2011). However, characterising species diets at a scale that reflects the complexity of diets (*i.e.,* spatio-temporal variations in diet composition) is challenging, and especially when relying on traditional methods such as direct observations or microhistology method (Hansson, 1970). Consequently, improving our capacity to access unbiased and taxonomically resolved diet data in a cost- and time-efficient way is crucial for accelerating basic knowledge about the trophic ecology of animals and conservation management.

Molecular analyses offer a new set of tools for accurately describing diet. DNA metabarcoding (Taberlet et al., 2018; Taberlet et al., 2012b) has quickly gained popularity thanks to its efficient and precise identification of food items based on their DNA sequences (Pompanon et al., 2012; Soininen et al., 2009; Valentini et al., 2009a). This method relies on the extraction of DNA from digested food remains in a dietary sample (*i.e.,* stomach content, regurgitate or faeces), its amplification with universal primers (Taberlet et al., 2012a; Valentini, Pompanon & Taberlet, 2009b), and then the sequencing of individual DNA molecules, identified by matching them to a sequence reference database. Metabarcoding is especially advantageous for complex and cryptic diets consisting of many taxonomically diverse taxa, as the method requires little prior knowledge about the system under study (De Barba et al., 2014). From a qualitative point of view, the capacity of dietary metabarcoding to detect even highly degraded, low abundant DNA, while providing high taxonomic resolution is particularly valuable, as it allows for accessing rare or taxonomically cryptic dietary taxa (Soininen et al., 2015; Sullins et al., 2018). DNA metabarcoding also has the potential to be quantitative—*i.e.,* to inform about the relative biomass proportions of ingested food (Newmaster et al., 2013; Willerslev et al., 2014). If realised, such a potential implies an essential breakthrough, as traditional methods such as microhistology are known to overestimate the proportion of taxa such as grasses compared to forbs (Anthony & Smith, 1974).

There are two approaches to achieve quantitative estimates of diet from metabarcoding data. One approach is to count the number of individuals/samples with recorded presence/occurrence of a given food item in the population of samples (Biffi et al., 2017; Xiong et al., 2016). The higher the count within the population, the larger the food item’s ecological contribution. In this way, the frequency of occurrence can provide quantitative information at the population level. However, this approach requires large sample sizes, especially if the diet is diverse. Moreover, even though an item may occur frequently in the populations, it may still be ecologically unimportant if most individuals consume it in relatively low quantities. The other approach is based on calculating the relative frequencies of sequence reads (*i.e.,* relative read abundance, RRA), where the number of reads is assumed to be proportional to the relative biomass of the corresponding food items (Deagle et al., 2010). In a recent review and meta-analysis, Lamb et al. (2019) show that relative read abundance and ingested food biomass correlate positively in some model systems (*e.g.*, Kartzinel et al., 2015; Newmaster et al., 2013; Nichols, Akesson & Kjellander, 2016; Thomas et al., 2014), but not in others (*e.g.*, Deagle et al., 2013; Elbrecht, Peinert & Leese, 2017; Hatzenbuhler et al., 2017; Piñol, Senar & Symondson, 2019). The highly variable correlation suggests that the proportion of reads should not be used as a proxy for diet proportions *a priori,* and that biases can arise from *e.g.*, DNA extraction (Majaneva et al., 2018) and DNA amplification (Bellemain et al., 2010). Also, bias from differential digestion of plants with different functional characteristics or digestibility can further increase variation in the quantitative output (Deagle et al., 2013; Leal et al., 2014; Nakahara et al., 2015; Thomas et al., 2014). Thus, validations specific to different animal groups are required for measuring DNA metabarcoding’s potential for the quantitative assessment of diets. However, food-item specific validations with information on ingested biomass proportions remain rare.

Small rodents are commonly used in ecological research because they make convenient model species (Hickman et al., 2017), and because of the fluctuating dynamics of many populations and important roles in food webs (Boonstra et al., 2016; Ehrich et al., 2017). Despite the increasing use of DNA metabarcoding for analysing their diets (Ozaki et al., 2018; Sato et al., 2018b; Soininen et al., 2015), no quantitative validations are available for small rodent systems, with earlier methodological studies mainly focusing on the comparison with alternative methods (Khanam et al., 2016; Soininen et al., 2009) or a molecular mock community (Iwanowicz et al., 2016). Here, we use experimental feeding trials to test the hypothesis that relative read abundance from rodent faecal samples closely reflects the ingested food biomass.

## Materials & Methods

We used the tundra vole *Microtus oeconomus*—a commonly studied herbivorous small rodent species with a circumpolar distribution—as our model species. Captive tundra voles were offered three experimental meal mixtures, each containing three plant species representing 60%, 30% and 10% of the total diet biomass. We collected vole faecal samples in each feeding trial and analysed them with a DNA metabarcoding approach using the universal *gh* plant primers of the *trnL* P6 loop region (Taberlet et al., 2007). Finally, we compared the plant biomass proportions from meal mixtures to the relative read abundance estimated by DNA metabarcoding.

### Feeding experiment

The feeding trials were conducted in accordance with Norwegian laws and regulations concerning experiments with live animals, which are overseen by the Norwegian Food Safety Authority (FOTS 15309, 15585). We obtained our experimental units, the tundra vole individuals (*n* = 9) from Håkøya, northern Norway (69.7°N, 18.5°E). The sample size was decided based on similar previous experiments (Deagle et al., 2013; Willerslev et al., 2014). All animals were juveniles trapped in July 2019 within their natural boreal meadow habitat, characterised by the frequent occurrence of the plant species we used as food in the experiment. Once trapped, individuals were kept close together in the same room, but in separate 40 × 30 × 25 cm cages. The room was naturally ventilated through large open windows, without heating or an artificial light scheme. We observed the animals intensively during the start of each experimental trial, and subsequently every 2 h throughout the experiment to refill food and to inspect animal health and welfare. Until the experiment started, we fed the animals *ad libitum* with fresh food items known to be eaten by small rodents in previous studies, including the plant species used in the meal mixtures. At this stage, regular observations showed that *Trifolium* was the most preferred item although no systematic measures were performed on the exact amount eaten. Voles were also offered small portions of the experimental meal mixtures for familiarisation.

We selected three plant species to compose the artificial meal mixtures offered to the animals –the white clover (*Trifolium repens* L., Fabaceae), the wavy hairgrass *(Avenella flexuosa* (L.) Drejer, Poaceae), and the pussy willow (*Salix caprea* L., Salicaceae). We selected these species because they (i) represent different functional groups (*i.e.,* forb, graminoid and shrub), (ii) are known to be preferred food items (Soininen et al., 2013); and (iii) are readily available in natural tundra vole habitats. We collected the plant material in separate bags from natural habitats. We cut the plants, ground them and stored them temporarily at 3 °C immediately after collection. We then extracted one subsample of ground plant biomass from each plant species individually (*n* = 3) to be used as reference. The remainder of the ground plant biomass was used for composing three meal mixtures (mock communities of fresh plant material) of the three plant species, to yield three dry weight biomass proportions, *i.e.,* 60%, 30% and 10% for each species (Fig. S1, Table S1). To make the mixtures, we used the dry weight ratios of plant subsamples that have been dried for 24 h at 80 °C. Plant biomass from the three plant species was mixed, homogenised into a porridge-like substance, and stored at 3 °C until use. This resulted in three meal mixtures named after their taxonomic contribution (*i.e.,* T10_S60_A30, T30_S10_A60 and T60_S30_A10, where T, S and A are abbreviations of the plant genus names). We also withdrew one sub-sample from each meal mixture before starting the feeding trials and stored it apart prior molecular analyses to disentangle biases arising from digestion from those arising from molecular analysis.

The three meal mixtures were offered *ad libitum* to all nine animals in three separate trials, though not all 9 × 3 samples were retrieved for analysis (see below). Arvicoline rodents have a fast metabolism, with 50% of the green plant particles passing through the alimentary tract in only 3–3.5 h, and with complete passage in 20 (Kostelecka-Myrcha & Myrcha, 1964) to 30 h (Lee & Houston, 1993). For hardly digestible items such as seeds, it may take up to twice this time to completely pass through the arvicoline alimentary tract (Kostelecka-Myrcha & Myrcha, 1964). Therefore, we decided to use only green plant material and allow the animals to feed on the same meal for 48 h before collecting faecal samples and starting a new trial using the same animals. Thus, the total length of the active experiment was six days. We collected an equal quantity of ten faecal pellets per animal and trial using forceps that has been sterilised with chlorine solution prior to each individual sampling. Faecal pellets were placed in filter paper bags and stored in plastic zip-lock bags, pre-filled with silica gel. We cleaned the cages with a chlorine solution prior to the experiment and between each trial in order to reduce the risk of cross-contamination. In a preceding pilot study, we also evaluated the risk of cross-contamination with environmental DNA coming from previous use of the same cages *via* the animals or the air by rubbing cages’ floor with sterile cotton tips. These analyses showed that contamination risk from the experimental setting was negligible (see Table S5). Consequently, we did not further consider this aspect.

We offered the meals to voles as a thoroughly mixed homogenous substance. Although the consistency of this mixture was unfamiliar to the animals, they ate it in all trials, except two of the individuals that ate very little of the second meal mixture T30_S10_A60. To prevent any animal welfare issues, these individuals were relieved from the trial with this meal. After their quick recovery they were included in the subsequent trial. In the end, all animals were euthanised by cervical dislocation. Since some of the samples were discarded after post-sequencing bioinformatic processing and data filtering, the final sample size was *n* = 23, including samples from meal mixtures (*n* = 3, one per meal mixture), individual plant subsamples (*n* = 3, one per plant species), and faecal samples (*n* = 17). Faecal samples were distributed between the meal mixtures so that mixtures T30_S10_A60 and T60_S30_A10 had *n* = 5, while meal mixture T10_S60_A30 had *n* = 7. We marked the samples with codes to process the samples and sequences blindly.

### Molecular analysis

DNA extractions from faecal pellets, individual plants and meal mixtures were performed by Sinsoma GmbH (Innsbruck, Austria) using the Biosprint 96 DNA Blood Kit (Qiagen) on a Biosprint 96 Robotic Platform (Qiagen). DNA extractions were carried out according to the manufacturer’s instructions, except that (1) the lysis step consisted in adding 250 µl lysis buffer (TES buffer: Proteinase K (20 mg/ml) 19:1) in each sample before vortexing and overnight lysis at 58 °C; and (2) DNA was eluted in 200 µl 1×  TE buffer. DNA extraction negative controls (water instead of DNA) were systematically included. As part of the standard procedure for quality control at Sinsoma, a subset of samples (all DNA negative controls and a random subset of DNA extracts from samples) were used to control for both possible cross-contaminations and the successful extraction of DNA. The general mitochondrial cytochrome oxidase I gene, COI (Folmer et al., 1994) was used for detection of animal DNA, while the nuclear internal transcribed spacer rDNA regions, ITS (Taberlet et al., 2007; Taberlet et al., 1991), was used for plant detection. As expected, the extraction negative controls were negative, and a positive band was observed for the extraction positive control, and thus these control samples were not included further.

As part of our feeding experiment, all samples were amplified with the *g* and *h* primers (Taberlet et al., 2007), targeting a highly variable length region (10–220 bp) from the P6 loop of the chloroplast *trnL* (UAA) intron in vascular plants. This primer set is particularly suitable for the analysis of highly degraded DNA (Clarke et al., 2020; Hollingsworth, Graham & Little, 2011; Särkinen et al., 2012; Schneider et al., 2021; Willerslev et al., 2014) due to its short amplicon size, highly variable gene region and conserved priming sites (Deagle et al., 2014; Taberlet et al., 2012a). The primer sequences are 5′-GGGCAATCCTGAGCCAA-3′and 5′-CCATTGAGTCTCTGCACCTATC-3′, respectively. We labelled the forward and reverse primers with unique 8–9 nucleotides sequence tags modified from Taberlet et al. (2018), allowing to distinguish individual samples following high-throughput sequencing. All PCR reactions were carried out in a total volume of 15  µL using the AmpliTaq Gold 360 PCR Master Mix (Thermo Fisher Scientific, Waltham, MA, USA), 0.4 µl/15 ml of bovine serum albumin (BSA; Sigma-Aldrich, USA), 0.5 µM of each primer and 2 µl of undiluted DNA. We initiated the PCR reaction by a denaturation step at 95 °C for 10 min, followed by 40 cycles consisting of denaturation at 95 °C for 30 s, annealing at 52 °C for 30 s, elongation at 72 °C for 1 min, and finally elongation at 72 °C for 7 min. We conducted three PCR replicates per sample. For each PCR-plate (*n* = 3), we included one PCR negative control (ultra-pure Milli-Q water instead of DNA) and one PCR positive control (*i.e.,* a mixture of six synthetic standard sequences with varying GC content, homopolymers, sequence length and concentrations, see Table S2). We visualised PCR products on a 1.5% gel electrophoresis before pooling and purifying PCR products using the QIAquick PCR Purification Kit (Qiagen). DNA concentration from purified amplicon pools was then quantified using a Qubit 2.0 fluorometer and the dsDNA HS Assay kit (Invitrogen, Life Technologies, USA). Purified pools were used for libraries preparation using the KAPA HyperPlus kit (Kapa Biosystems, USA), and sequenced (2 × 150 bp paired-end reads) on a HiSeq 4000 machine (Illumina, USA) following manufacturer’s instructions at the Norwegian Sequencing Centre. The sequencing was carried out in two separate runs, and we merged the sequence reads data from both runs during the bioinformatic filtering process.

### Bioinformatics

We carried out bioinformatic analyses using the OBITools bioinformatics pipeline (Boyer et al., 2016) on the Norwegian high-performance computing cluster Sigma2. All commands referred to in this paragraph are from the OBITools python package (http://metabarcoding.org/obitools). We processed the raw data in the following order: (i) merging of the forward and reverse reads (with minimum quality score threshold of 40) with the *illuminapairedend* command, (ii) removing low quality reads (with alignment score less than 50) with the *obigrep* command, (iii) assigning sequences to samples based on identification tags with the *ngsfilter* command (*i.e.,* demultiplexing, which also required perfect match between the tag and the target sequence, and a maximum of 2 bp mismatch between the primers and the target sequence), (iv) merging strictly identical sequences into single molecular operational taxonomic units (*i.e.,* MOTUs) with the *obiuniq* command, (v) removing short (less than 10 bp) and rare (occurring with less than 10 copies in the entire dataset) sequences with the *obigrep* command, and (vi) flagging erroneous sequences owing to PCR and/or sequencing with the *obiclean* command.

We created a local reference database from the reference library “ArctBorBryo” (Soininen et al., 2015, Sønstebøet al., 2010; Willerslev et al., 2014) and the European Nucleotide Archive nucleotide library (EMBL, release 143, accessed in April 2020) with the *ecoPCR* program (Bellemain et al., 2010; Ficetola et al., 2010). Finally, we compared the reference database to the sequences in our data, assigning each sequence to a taxon with the *ecoTag* program (Pegard et al., 2009).

Further data filtering, visualisation and analyses were conducted with the R software version 4.0.3 (R Core Team, 2020) using ROBITools package (http://metabarcoding.org/obitools). To start with, all MOTUs flagged as erroneous by *obiclean* (OBITools, Boyer et al. (2016)) were removed. Afterwards, we filtered out PCR outliers based on the comparison of Euclidean distances of PCR replicates with their average, and with the distribution of pairwise dissimilarities between all average samples. PCR replicates flagged as outliers were iteratively removed from the dataset. We averaged the number of reads per MOTU in the remaining PCR replicates for each sample. At this stage, all remaining MOTUs whose relative frequency in a PCR was inferior to 1% were filtered out. Next, only MOTUs with identity match ≥85% to sequences in the reference library were kept for further analyses. Due to the known and taxonomically very restricted diet, we only kept relevant MOTUs to estimate proportions were the best identified taxonomic level. Finally, we normalised the sequence read abundances by dividing the number of reads for each MOTU by the total number of reads within each sample.

### Statistical analyses

We assessed the quantitative accuracy of dietary metabarcoding by using a multivariate regression model that establishes a linear function between the multiple compositional outcomes (responses) and compositional predictors (Fiksel, Zeger & Datta, 2021). Here we used the composition of relative read abundance of each of the three plant species (RRA from faeces or meal mixtures) as response variables and the expected plant species composition (*i.e.,* known biomass composition) as predictor variables. This type of compositional analysis accounts for the fact that an increase in one taxon’s proportion will force a decrease in other taxon(s) proportion within the same sample (Alenazi, 2019; Chen, Zhang & Li, 2017; Fiksel, Zeger & Datta, 2021). The model allows, without transformation, for direct interpretation of the relationship between expected and observed compositions through a Markov transition matrix “B” based on the estimated regression coefficients. Since both the predictor and the response variables are compositions, the regression coefficients (*i.e.,* the matrix B) is constrained to non-negative values in the range of 0 to 1, and each row of the matrix sums to 1. These coefficients describe how the outcome composition would change in relation to a change in the predictor composition. Values close to one along the diagonal indicate a high correlation between the outcome and the predictor compositions.

We computed the regression coefficient (B matrices) and tested for overall linear independence *via* permutation tests (using an α-level of 0.05) with the codalm package (Fiksel & Datta, 2020; Fiksel, Zeger & Datta, 2021) in the R software (R version 4.0.3). To assess the model’s goodness of fit, we plotted the predicted values *versus* the observed RRA of the faecal samples, using leave-one-out cross-validation (LOOCV) (Fiksel, Zeger & Datta, 2021, see Fig. S2).

## Results

### Sequencing output

This experiment’s samples were multiplexed with samples from another project, so the number of sequences is only known after identifying the sequences with their sample tags during the *ngsfilter* step, resulting in 2,080,764 sequences (Table S3 gives step-by-step details of read/sequence counts during the bioinformatics workflow). After data cleaning and merging of the PCR replicates, 1,477,342 reads were assigned to faecal samples. Of the nine MOTUs in the final dataset, one was only assigned to the family level, one to the subfamily/tribe, and the remaining seven were assigned to the species/genera (Table S4). Four of these MOTUs were identified at a lower taxonomic level using BLAST search (Altschul et al., 1990). For details on the filtering steps, see Table S3. The final filtered dataset, as well as the raw high-throughput sequences, are available in Dataverse (https://doi.org/10.18710/HJAVSN).

### Taxonomic assignments

We assigned most of the cleaned sequence reads (>97%) to the three expected plant species. We retrieved all three positive PCR control replicates after sequencing, and we only detected the synthetic sequences in their corresponding sample. None of the negative control samples (*i.e.,* samples without DNA) passed the data filtering steps. The synthetic sequences added as a positive PCR control had varying amplicon length (30-60 bp) and varying GC content (20–40%), and close to the expected log-linear relationship (Fig. S3). The MOTUs retrieved from the faecal samples, individual plants and meal mixtures (Table S4) had similar GC-content composition (13–21%) and amplicon length (45–56 bp) as the amplified positive control sequences with little variation among MOTUs.

### Analyses of plant items and meal mixtures

In the subsampled plant material from the single plant taxa, the mean number of reads per sample ranged between 10,166 and 29,438, from Fabaceae being the least amplified to Salicaceae with almost three times more reads retrieved (note that these plants were sequenced in separate PCR replicates). From each single plant sample, we only detected MOTUs corresponding to the respective taxonomic family of the plant sequenced—*i.e.,* one MOTU per plant sample. In the *Avenella flexuosa* sample, we also detected a second MOTU best identified as *Festuca* sp., but which was amplified in much smaller proportion. As this plant material was collected in the field, a non-targeted species might thus have been accidentally included.

**Figure 1:**
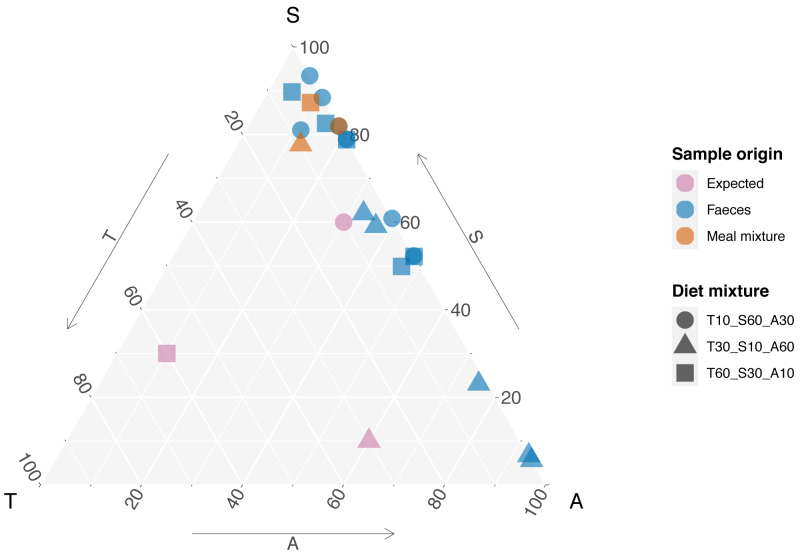
Expected composition of diet mixtures and relative read abundance (RRA) acquired from meal mixtures and rodent faeces. Edges of the triangle represent the three species proportions, T, S, and A short for the plant species *Trifolium repens, Salix cabrea,* and *Avenella flexuosa*, respectively. Each tip of the triangle represents 100% for the given species and 0% for the other species. Symbols for expected composition are based on known biomass proportions, whereas symbols for meal mixture and faeces RRA represent one sample each (*i.e.,* mean across three PCR replicates). Note that the symbols are plotted transparency, and stronger colours thus indicate several overlapping data points.

**Figure 2:**
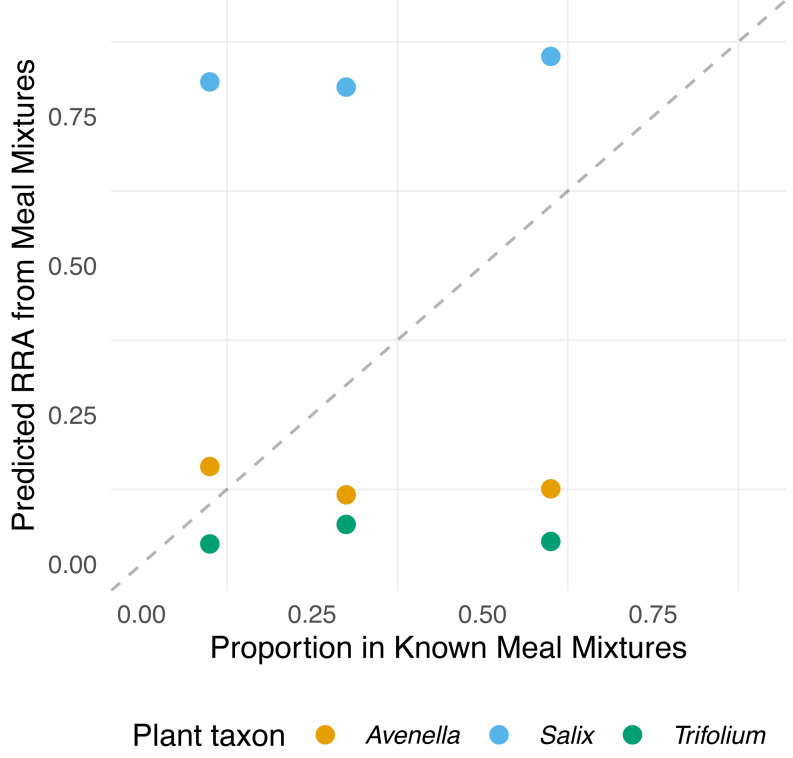
Relationship between expected proportions of the known diet and predicted proportions of food items in meal mixtures. Each point is based on model predictions from the compositional regression. The dashed line shows 1:1 relationship.


In the meal mixtures, we detected only the three expected MOTUs, with the exception of one sample (T10_S30_A60), from which *Trifolium* MOTU was missing. All three samples had high RRA of *Salix* and low RRA of *Trifolium* (Fig. S1, filtered dataset at Dataverse). We found no evidence for a relationship between the RRA and expected composition (permutation test for linear independence *p* = 0.49, Figs. 1 and 2, Fig. S4). The estimated B-matrix

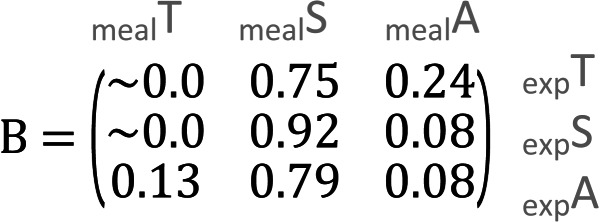


showed that the outcome composition of Salix was well predicted by its proportion in the predictor composition (corresponding to 0.92 on the diagonal). However, the proportions of Trifolium and Avenella in the outcome compositions were little related to their proportions in the predictor composition (corresponding to 0.0 and 0.08 on the diagonal).

### Dietary analyses

All faecal samples contained MOTUs belonging to two of the expected plant species, *Salix caprea* and *Avenella flexuosa*, but several samples did not retain reads from *Trifolium* after data processing. We identified five unexpected MOTUs that were seemingly contaminants (Table S4). Two of these potentially originate from the food given to the voles before the experiment (*Maleae* sp. found in three samples, total 0.2% of the reads, and *Avena* sp. found in 1 sample, total 0.1% of the reads). Additionally, we identified the MOTU best representing *Festuca* in six of the faeces samples. Due to the possibility for field sampling error, we therefore merged *Festuca* (4% of the total composition) with *Avenella flexuosa* in the quantitative assessment (see above).

**Figure 3:**
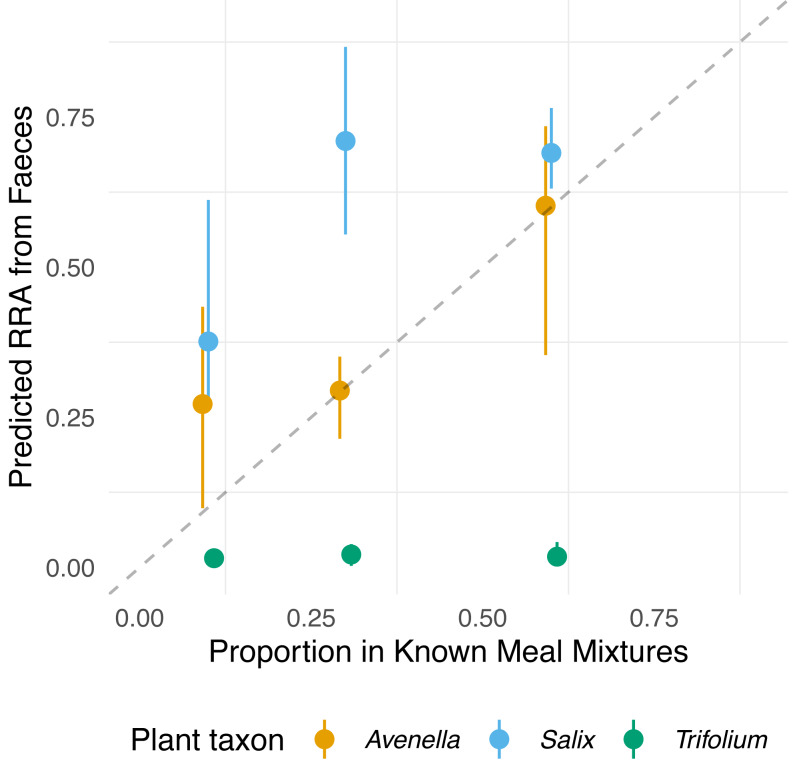
Relationship between expected proportions of the known diet and predicted proportions of food items in vole diets. Each point is based on model predictions from the compositional regression, with the bootstrapped upper/lower confidence intervals boundaries around each prediction. The dashed line shows 1:1 relationship.


The compositions of RRA from faecal samples had a weak or moderate relationship with the expected composition (Fig. 3). In particular, all faecal samples had meagre *Trifolium* proportions compared to the expected proportions (Figs. 1, 3 and Fig. S4). However, we found evidence for a positive linear relationship between expected and observed values for two of the species. The estimated B-matrix

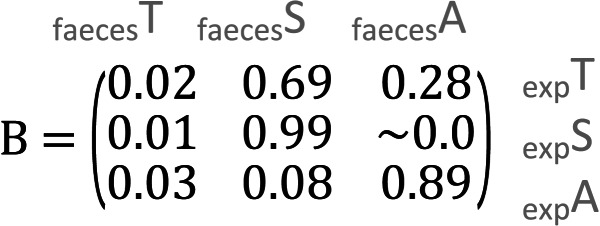


showed that the proportions of *Salix* and *Avenella* in the outcome compositions were well predicted by their proportions in the predictor compositions (*i.e.,* 0.99 and 0.89 on the diagonal). The same was not true for *Trifolium* (0.02 on the diagonal). See Table 1 for confidence intervals on parameter estimates. The permutation test (*p* = 0.009) yielded evidence for a linear dependence between the expected compositions and RRA. The predicted values obtained through the leave-one-out cross-validation procedure indicated that the model fit was reasonably good (Fig. S2).

**Table 1:** Confidence intervals of the parameter estimates. Values in the B-matrices below represent 95% confidence intervals obtained from bootstrapping around each prediction of the compositional regression.


**(A) Meal ∼ Expected (95% confidence intervals)**
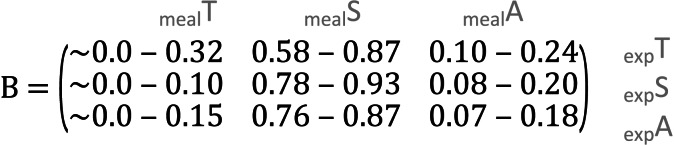
**(B) Faeces ∼ Expected (95% confidence intervals)**
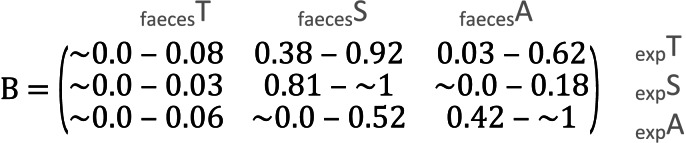


## Discussion

We investigated the relationship between diet composition obtained by DNA metabarcoding of vole faeces and the consumed food’s composition. This is one of few studies relating ingested biomass to quantitative metabarcoding analysis of animal faeces, and as far as we know, the only one on herbivorous small mammals. We found that the expected and observed proportions of plant species in vole faeces were correlated for two out of three plant species. However, the third plant species had consistently low proportion and blurred the overall relationship between expected and observed diet compositions. Our results suggest that a certain degree of caution is necessary when making conclusions on the species’ ecology based on relative read abundances (RRA) estimated from DNA metabarcoding data.

We found an overall poor consistency between the RRA of meal mixture samples and the actual composition of the meals. We find potential biases with food item-specific DNA retrieval (Deagle & Tollit, 2007), as the main issue was that one of the three species in our study (*Trifolium*) performed consistently poor. It had a very low recovery in the meal mixtures, with several samples containing no reads at all. Furthermore, the number of retrieved *Trifolium* read abundance from the samples containing only this species were considerably lower than for corresponding samples for other species. This pattern in *Trifolium* detectability is puzzling since previous studies did not report bias related to this species (Willerslev et al., 2014). One plausible explanation for our results is the small sample size, as we analysed a single sample of each meal mixture. Therefore, we recommend a more substantial sub-sampling of meal mixtures used in such feeding trials. An additional advantage with a higher number of sub-samples is the possibility to use RRA from meal mixtures to generate correction factors (*sensu* Thomas et al. (2014)) to control for potential differences in digestion of food items. However, the practicality of such correction factors in generalist herbivores such as voles is to be further demonstrated, as they often include a large number of food plant species in their diet (Soininen et al., 2013). Also, as ecological studies of diets most often are based on several individuals averaged together (as opposed to a single sampling or sample site), some of the benefits of using correction factors are already incorporated (Thomas et al., 2016). Rather, in such study systems, it may be better to aim at understanding the underlying processes that inflict food-item specific biases.

A similar poor consistency between RRA of meal mixture samples and the actual composition of the meals has previously been described (Deagle & Tollit, 2007; Deagle et al., 2010). Unfortunately, our study design do not allow us to pinpoint the exact mechanisms behind the observed limited retrieval of *Trifolium*. However, the consistently low detection of *Trifolium*, irrespective of the sample type, could indicate a systematic bias, potentially due to lower chloroplast DNA content (Soltis, Soltis & Milligan, 1992), although not much information is available about the chloroplast numbers variation in *Trifolium* comparatively to other plant taxa such as *Poaceae* or *Salicaceae* for example. However, the nuclear DNA content in *Trifolium* has been reported to have large variation (Vižintin & Bohanec, 2008; Vizintin, Javornik & Bohanec, 2006), *T. repens* differing up to 21% between lineages/varieties (Campbell, Caradus & Hunt, 1999; Vižintin & Bohanec, 2008). Nevertheless, and regardless of possible variations in chloroplast content, one of the advantages of DNA metabarcoding is its ability to retrieve even very small proportions of DNA, as exemplified by other feeding experiments using *Trifolium* (Willerslev et al., 2014).

Another potential source of bias can arise from DNA amplification. The *gh* gene marker used in our study offers well-conserved priming sites across lineages and should be well adapted for amplifying our target species (Baksay et al., 2020; Taberlet et al., 2018). Based on earlier DNA metabarcoding studies using the *gh* primers, there are seemingly no issues with differential DNA extraction or amplification of *Trifolium* (Nichols, Akesson & Kjellander, 2016; Pornon et al., 2016; Willerslev et al., 2014). Our analyses of single-species samples indicate that the low amplification of *Trifolium* did not depend on which other plants were present in the samples. Furthermore, as positive control standards showed close to the expected log-linear relationship, we have no indication of issues related to amplicon length and GC content. Finally, the *Trifolium* reads abundance falls within the range of variation for the (successfully amplified) positive control standards. Moreover, the priming sites of the *Trifolium* MOTUs we retrieved had no mismatches as compared to the *gh* primer pair. Yet, these exploratory results are based on low sample sizes, and our study do not allow for a more precise assessment of the mechanisms behind this species’ low amplification success. Using quantitative PCR (qPCR) to calculate amplification efficiency would have avoided speculation on this issue, and we recommend considering this in future feeding experiments.

The weak relationship between diet inferred from faeces and the expected plant composition could also be explained by differential digestion of plants (Deagle et al., 2010). The amplification of *Trifolium* was, however, problematic independent of whether samples had been digested by voles or not. We also controlled for some influential sources of variation in digestion (*i.e.,* we offered only plant leaves and kept the animals on the same meal mixture for an extended period). While DNA traces of the previous meals might still be present in the voles’ digestive system, we did our best to minimize their effect in our design. In the absence of studies on the DNA decay of food in the digestive tracts of rodents (but see Schattanek et al. 2021), we selected a conservative time-frame for the different feeding trials that is compatible with the digestion of diet’s hard remains. Furthermore, the RRA of the two other plants (*Salix* and *Avenella*) seemed to correlate well in meal mixtures and faecal samples. We thus conclude that the impact of digestion on our results must be rather small.

Differences in the sequence reads number can to some extent be corrected for in the bioinformatic processing by using proportions or rarefying to normalize data, and contrasting methods are currently a topic of discussion (McKnight et al., 2019; McMurdie & Holmes, 2014). During data processing, we also attempted a stricter filtering of the faecal samples compared to what is currently presented in the main results. Stricter filtering resulted in discarding more samples, but interestingly, the reduction was not equal between meal compositions. Faecal samples resulting from diets with high proportions of *Trifolium* were filtered more strongly than samples from diets with low proportions of *Trifolium*. This resulted in an over-representation of samples where proportion of *Trifolium* was both expected and observed to be low, thus strengthening the correlation estimates. This shows the importance for consistent *a priori* decisions for the bioinformatic processing.

Despite the growing use of DNA metabarcoding for analysing small rodent diets (Ozaki et al., 2018; Sato et al., 2018; Soininen et al., 2015), our study was the first to compare the quantitative reliability of the method using known meals fed to rodents. We found a correlation between observed and expected diets for only some of the plant species, and the overall observed diet composition did not reflect the expected composition well. Even moderate but systematic deviations in retrieval greatly reduce a correlation between observed and expected compositions in a mixture containing only few taxa (like our three-species meals). Thomas et al. (2016) suggest that diets with a higher number of species are less biased as different DNA molecules are more equally represented during the PCR, consequently reducing biases such as self-annealing (for the very abundant molecules). The correlation between observed and expected compositions may thus be weaker for meal mixtures containing only few taxa compared to meal mixtures containing more taxa. This also gives hope that sampling of more complex mixtures and natural diets of generalist herbivores in general provide more robust output than the one observed here. Moreover, independently of the number of species, the composition itself (*i.e.,* having extreme proportions) influences the correlation (Deagle et al., 2019). This is exemplified in Willerslev et al. (2014) with proportions of both 0% and 100% in their composition, showing a strong correlation in a two-species system. Consequently, previous studies of complex diets give relatively consistent compositions across different methods for rodents (Khanam et al., 2016; Soininen et al., 2009) and other herbivorous mammals (Newmaster et al., 2013), or for mock communities (Iwanowicz et al., 2016). However, we find it likely that DNA-based analyses of also diets composed on many food items will be hampered if any dominant food item has the same problematic issues as we have identified for *Trifolium.*

During the last 10 years, DNA metabarcoding has proved valuable to expand the understanding of trophic interactions. Our findings for small rodents add to the growing number of assessments of different taxa showing that caution is necessary when drawing ecological conclusions from sequence reads count data. Based on our experience with these analyses, we have two main messages for future developments of DNA metabarcoding diet studies. *First*, comparisons between observed and expected diets will benefit from using the direct regression approach of Fiksel, Zeger & Datta (2021), where both response and predictor variables are compositional. Although this method does not require transformation and is easier to interpret, it does not allow for further covariates. Most compositional analysis methods currently available have similar or other shortcomings, which would require further developments to overcome. *Second*, while DNA metabarcoding can give quantitative results, they are unlikely to be perfect. The metabarcoding process involves many steps, each of which is susceptible to errors and biases (cf. Alberdi et al. (2018)). Our study is only the first step aiming at testing whether a positive correlation between observed and expected diet compositions exists for rodents. Currently, DNA metabarcoding represents the most accessible and cheap DNA-based option for diet analysis. We therefore see the advantage of studies that aim to pinpoint and better understand the mechanisms of potential biases, as no such study exists on the matter, in neither rodents and herbivores.

##  Supplemental Information

10.7717/peerj.11936/supp-1 Supplemental Information 1 The composition of relative read abundance (RRA) in meal mixtures and faeces of rodents that were fed these mixtures To the right, identity of meal mixture. The plant species *Trifolium repens, Salix cabrea* and* Avenella flexuosa* are shortened to T, S and A, respectively, and numbers refer to the proportion of each species in the given meal mixture. To the left, sample type. *Expected* refers to known biomass composition of the meal mixture, *meal mixture* to RRA of the meal mixture, and *individuals* to the sampled rodent individuals (one trial per individual per meal mixture).

10.7717/peerj.11936/supp-2 Supplemental Information 2 Model fit for multivariate regression predicting observed compositions of plant taxa in rodent faeces The expected values (predicted from model) for each of the faecal sample compositions, obtained by leave-one-out cross-validation (LOOCV), are plotted against the observed RRA of the faecal samples. The distance between the dots and the dashed line represent the prediction error from the LOOCV (*i.e.,* goodness of fit). The dashed line shows 1:1 relationship.

10.7717/peerj.11936/supp-3 Supplemental Information 3 Number of observed reads of the synthetic DNA sequences used as positive control The sequences are arranged on the *X*-axis according to the (decreasing) proportion they composed of the analysed sample. For each sequence, the log across means is plotted. N per sequence is 1.

10.7717/peerj.11936/supp-4 Supplemental Information 4 Compositional regression coefficients Panel (A) regression of known plant composition of meal mixture components (predictor) on observed RRA in meal mixtures (response), panel (B) shows similar regression with observed RRA in vole faeces as response variable. The points represent species-specific regression coefficients, *i.e.,* the effect of a given species proportion on its observed RRA. They correspond to the effect of increasing the species proportion to one, while other species proportions are reduced to zero. Edges of the triangle represent the three species proportions. For a regression where expected composition is a strong predictor of the observed composition, the coefficient points are thus expected to be each in that tip of the triangle where the species proportion is one. The dotted lines represent 95% confidence intervals obtained from bootstrapping the results of the compositional regression.

10.7717/peerj.11936/supp-5 Supplemental Information 5 Characteristics of the meal mixtures used in the feeding experiment Note that the relative dry weight is similar for all plants. All proportions refer to the given plant species proportion of the given meal mixture. All values in grams.

10.7717/peerj.11936/supp-6 Supplemental Information 6 Summary of the synthetic DNA standards used as positive controls These DNA standards were used as PCR positive control mock community with the *gh* primer pairs (Taberlet et al., 2007) comprising a mixture of 6 standards, whose differences in sequence length (excluding priming sites), % GC content and relative concentration are presented below.

10.7717/peerj.11936/supp-7 Supplemental Information 7 Statistics of sequences and reads during data processing Columns number of reads and number of sequences refer to the remaining reads and sequences after each step of data processing. Read numbers denoted with * belong to a dataset that combined data from several studies, approximately 50% of samples not belonging to other than this study.

10.7717/peerj.11936/supp-8 Supplemental Information 8 Summary of MOTUs identified in the final filtered dataset Nine taxa were detected in the filtered samples (*N* = 23 samples of plants, meal mixtures, and faeces). The table is sorted by the number of samples (N) where each MOTU was detected. RRA refers to the relative read abundance in the entire filtered dataset.

10.7717/peerj.11936/supp-9 Supplemental Information 9 Evaluation of cross-contamination in a preceding pilot study Mean reads per sample/MOTU from sequenced swipes of floor using cotton swabs, and sequenced faeces collected from the same floor. Non-meal MOTU are MOTUs that represent taxon that does not match plant items that were offered to the animals in the meal mixtures. Note that these data are from a pilot study, and not from the same faeces samples as in the main study. The process for data extraction and bioinformatics was the same as in the main study.

10.7717/peerj.11936/supp-10 Supplemental Information 10 ARRIVE 2.0 Checklist

## Additional Information and Declarations

### Data Availability

The following information was supplied regarding data availability: Raw read data are available at the European Nucleotide Archive (ENA): PRJEB43213. Raw read data and processed data are available at Dataverse: Magne Neby, 2021, “Replication Data for: Issues of under-amplification in quantitative DNA metabarcoding weaken the inference about diet of the tundra vole Microtus oeconomus”, https://doi.org/10.18710/HJAVSN, DataverseNO, V1.
