# Supplementary figures and images for: Issues of under-representation in quantitative DNA metabarcoding weaken the inference about diet of the tundra vole Microtus oeconomus
Source: PeerJ. 2021 Aug 26;9:e11936. doi: 10.7717/peerj.11936 (PMC8403475; doi:10.7717/peerj.11936)

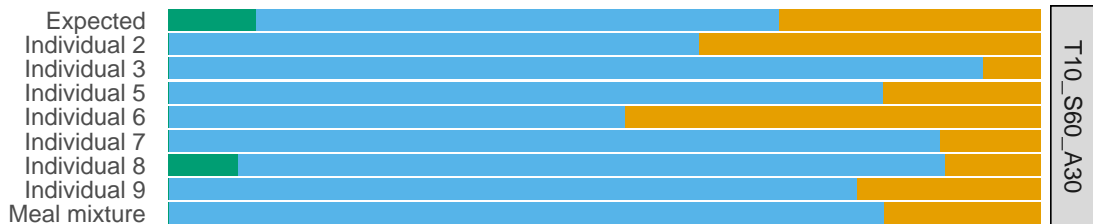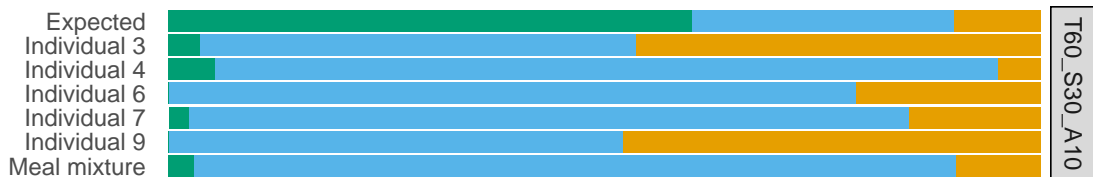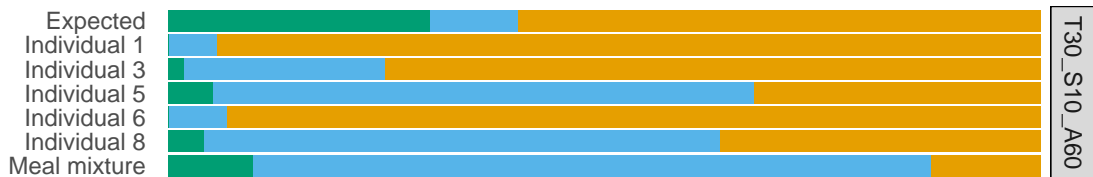

Sample compositions

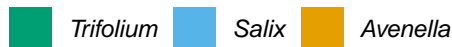

Supplement: Supplemental Information 1 — To the right, identity of meal mixture. The plant species Trifolium repens, Salix cabrea and Avenella flexuosa are shortened to T, S and A, respectively, and numbers refer to the proportion of each species in the given meal mixture. To the left, sample type. Expected refers to known biomass composition of the meal mixture, meal mixture to RRA of the meal mixture, and individuals to the sampled rodent individuals (one trial per individual per meal mixture). [file peerj-09-11936-s001.pdf]

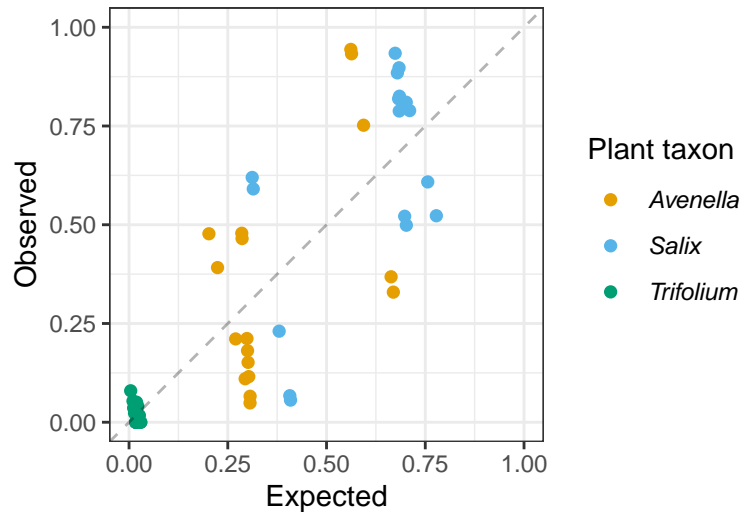

Supplement: Supplemental Information 2 — The expected values (predicted from model) for each of the faecal sample compositions, obtained by leave-one-out cross-validation (LOOCV), are plotted against the observed RRA of the faecal samples. The distance between the dots and the dashed line represent the prediction error from the LOOCV (i.e., goodness of fit). The dashed line shows 1:1 relationship. [file peerj-09-11936-s002.pdf]

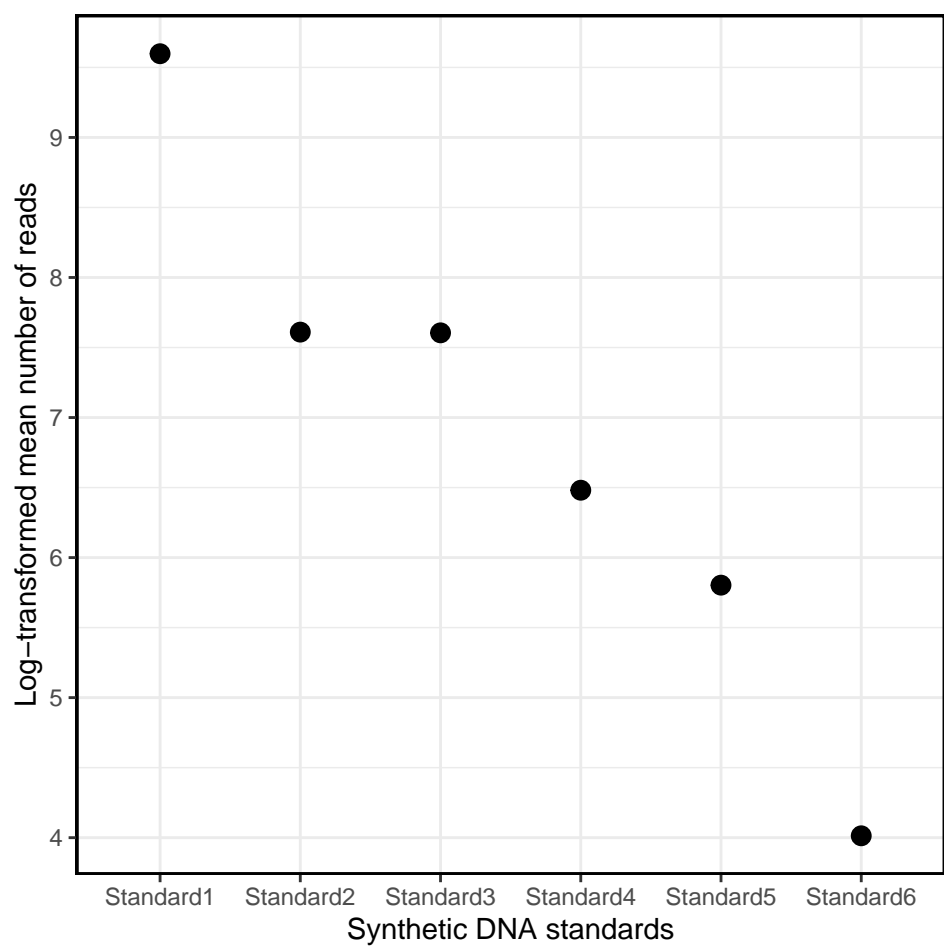

Supplement: Supplemental Information 3 — The sequences are arranged on the X-axis according to the (decreasing) proportion they composed of the analysed sample. For each sequence, the log across means is plotted. N per sequence is 1. [file peerj-09-11936-s003.pdf]

A

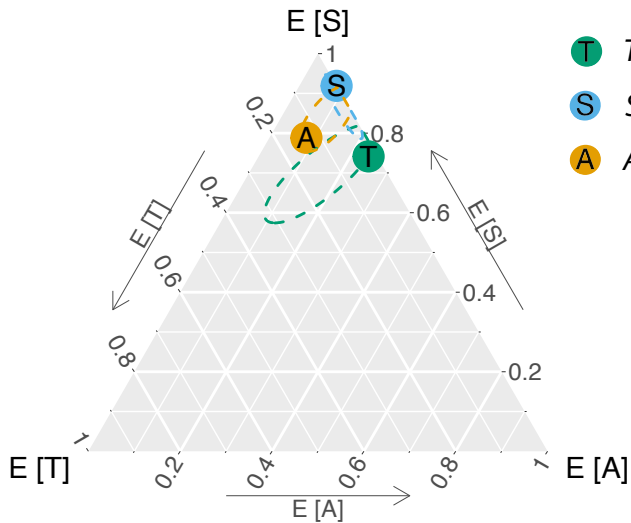

B

- *Trifolium*
- *Salix*
- *Avenalla*

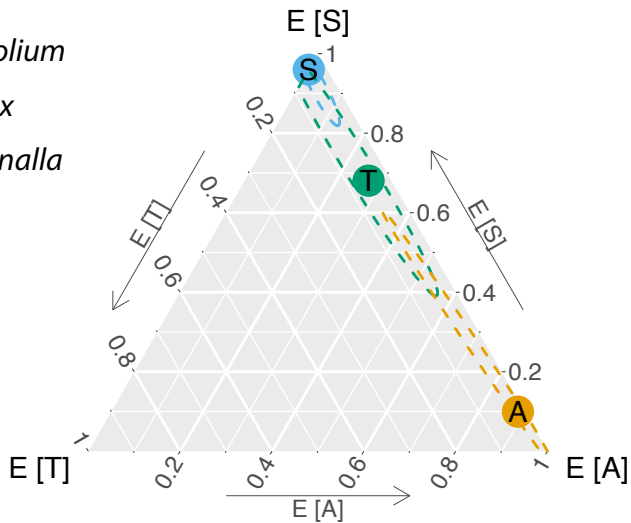

Supplement: Supplemental Information 4 — Panel (A) regression of known plant composition of meal mixture components (predictor) on observed RRA in meal mixtures (response), panel (B) shows similar regression with observed RRA in vole faeces as response variable. The points represent species-specific regression coefficients, i.e., the effect of a given species proportion on its observed RRA. They correspond to the effect of increasing the species proportion to one, while other species proportions are reduced to zero. Edges of the triangle represent the three species proportions. For a regression where expected composition is a strong predictor of the observed composition, the coefficient points are thus expected to be each in that tip of the triangle where the species proportion is one. The dotted lines represent 95% confidence intervals obtained from bootstrapping the results of the compositional regression. [file peerj-09-11936-s004.pdf]
