# Supplementary material for: Issues of under-representation in quantitative DNA metabarcoding weaken the inference about diet of the tundra vole Microtus oeconomus
Source: PeerJ. 2021 Aug 26;9:e11936. doi: 10.7717/peerj.11936 (PMC8403475; doi:10.7717/peerj.11936)
Supplement: Supplemental Information 5 — Note that the relative dry weight is similar for all plants. All proportions refer to the given plant species proportion of the given meal mixture. All values in grams. [file peerj-09-11936-s005.docx]

| Meal mixture | T60_S30_A10 | | |
| --- | --- | --- | --- |
| Plant species | *Trifolium repens* | *Salix caprea* | *Avenella flexuosa* |
| Dry weight proportion | 60.0 | 30.0 | 10.0 |
| Dry weight (total in meal) | 300.0 | 150.0 | 50.0 |
| Fresh weight (total in meal) | 574.4 | 287.7 | 93.0 |
| Fresh weight proportion | 60.1 | 30.1 | 9.7 |
|  |  |  |  |
| Meal mixture | **T30_S10_A60** | | |
| Plant species | *Trifolium repens* | *Salix caprea* | *Avenella flexuosa* |
| Dry weight proportion | 30.0 | 10.0 | 60.0 |
| Dry weight (total in meal) | 150.0 | 50.0 | 300.0 |
| Fresh weight (total in meal) | 287.2 | 95.9 | 558.2 |
| Fresh weight proportion | 30.5 | 10.2 | 59.3 |
|  |  |  |  |
| Meal mixture | **T10_S60_A30** | | |
| Plant species | *Trifolium repens* | *Salix caprea* | *Avenella flexuosa* |
| Dry weight proportion | 10.0 | 60.0 | 30.0 |
| Dry weight (total in meal) | 46.7 | 280.0 | 140.0 |
| Fresh weight (total in meal) | 89.4 | 537.1 | 260.5 |
| Fresh weight proportion | 10.1 | 60.6 | 29.4 |
