# Supplementary material for: Issues of under-representation in quantitative DNA metabarcoding weaken the inference about diet of the tundra vole Microtus oeconomus
Source: PeerJ. 2021 Aug 26;9:e11936. doi: 10.7717/peerj.11936 (PMC8403475; doi:10.7717/peerj.11936)
Supplement: Supplemental Information 6 — These DNA standards were used as PCR positive control mock community with the gh primer pairs (Taberlet et al., 2007) comprising a mixture of 6 standards, whose differences in sequence length (excluding priming sites), % GC content and relative concentration are presented below. [file peerj-09-11936-s006.docx]

| Standard | Sequence (5’-3’) | Size (bp) | GC content (%) | Dilution factor |
| --- | --- | --- | --- | --- |
| 1 | taagtctcgcactagttgtgacctaacgaatagagaattctataagacgtgttgtcccat | 60 | 40 | 1 |
| 2 | gtgtatggtatatttgaataatattaaatagaatttaatcaatctttacatcgcttaata | 60 | 20 | 0.5 |
| 3 | cacaatgctcggtaactagaagcatttgta | 30 | 40 | 0.25 |
| 4 | attgaatgaaaagattattcgatatagaat | 30 | 20 | 0.125 |
| 5 | agaacgctagaatctaagatggggggggggatgagtaagatatttatcagtaacatatga | 60 | 40 | 0.0652 |
| 6 | atttttgtaactcattaacaattttttttttgatgtatcataagtactaaactagttact | 60 | 20 | 0.03125 |
