# Supplementary material for: Issues of under-representation in quantitative DNA metabarcoding weaken the inference about diet of the tundra vole Microtus oeconomus
Source: PeerJ. 2021 Aug 26;9:e11936. doi: 10.7717/peerj.11936 (PMC8403475; doi:10.7717/peerj.11936)
Supplement: Supplemental Information 7 — Columns number of reads and number of sequences refer to the remaining reads and sequences after each step of data processing. Read numbers denoted with * belong to a dataset that combined data from several studies, approximately 50% of samples not belonging to other than this study. [file peerj-09-11936-s007.docx]

| **OBITools  command** | **Function** | **Number  of reads** | **Number of  sequences** |
| --- | --- | --- | --- |
| illuminapairedend | Align forward and reverse reads to reconstruct amplicons | 29928133* | NA |
| obigrep | Remove badly aligned/reconstructed sequences | 29865796* | NA |
| ngsfilter | Assign sequences to samples (demultiplexing) | 2080764 | NA |
| obigrep | Remove between-sample chimeras | 1892055 | NA |
| obiuniq | Group identical sequences into a unique sequence with count of sequence read per sample (dereplication) | 1892055 | 11384 |
| obigrep | Remove sequences with low frequency (<10) and sequences shorter than 10 bp | 1876336 | 992 |
| obiclean | Tag PCR and sequencing errors | 1876336 | 992 |
| ecotag | Assign sequences to taxa | 1876336 | 992 |
|  | Only keep sequences matching 95% with Arctborbryo database | 1876336 | 992 |
|  | Extract obiclean data and only keep MOTUs head at least once | 1777527 | 79 |
|  | Only keep the sequence that are less than 2 standard deviations, and sequence reads present in >1 PCR repeats per sample, and more than max | 1477342 | 79 |
|  | Only keep main MOTUs per taxonomic annotation | NA | 24 |
|  | Only keep Spermatophyta | NA | 23 |
|  | Only keep sequences corresponding to ≥1% presence in samples | NA | 9 |
