# Supplementary material for: Issues of under-representation in quantitative DNA metabarcoding weaken the inference about diet of the tundra vole Microtus oeconomus
Source: PeerJ. 2021 Aug 26;9:e11936. doi: 10.7717/peerj.11936 (PMC8403475; doi:10.7717/peerj.11936)
Supplement: Supplemental Information 8 — Nine taxa were detected in the filtered samples (N = 23 samples of plants, meal mixtures, and faeces). The table is sorted by the number of samples (N) where each MOTU was detected. RRA refers to the relative read abundance in the entire filtered dataset. [file peerj-09-11936-s008.docx]

| **Order** | **Family** | **Subfamily/tribe** | **Genus/Species** | **Amplicon length (bp)** | **GC content (%)** | **N** |
| --- | --- | --- | --- | --- | --- | --- |
| *Malpighiales* | *Salicaceae* | *Saliceae* | *Salix caprea** | 56 | 15 | 21 (0.588) |
| *Poales* | *Poaceae* | *Poeae* | *Avenella flexuosa* | 53 | 21 | 21 (0.301) |
| *Fabales* | *Fabaceae* | *Trifolieae* | *Trifolium sp* | 51 | 13 | 10 (0.062) |
| *Poales* | *Poaceae* | *Pooideae* | *Festuca sp* | 53 | 20 | 7 (0.025) |
| *Pinales* | *Pinaceae* | *Pinoideae* | *Pinus sp* | 45 | 19 | 4 (0.016) |
| *Rosales* | *Rosaceae* | *Amygdaloideae* | *Maleae sp* | 51 | 13 | 3 (0.004) |
| *Poales* | *Poaceae* | *Poeae* | *Avena sp** | 48 | 21 | 1 (0.001) |
| *Asterales* | *Asteraceae* | *Heliantheae** |  | 50 | 16 | 2 (0.002) |
| *Poales* | *Poaceae* |  |  | 53 | 20 | 1 (0.001) |

*Best description from BLAST based on the MOTU sequence
