# Supplementary material for: Issues of under-representation in quantitative DNA metabarcoding weaken the inference about diet of the tundra vole Microtus oeconomus
Source: PeerJ. 2021 Aug 26;9:e11936. doi: 10.7717/peerj.11936 (PMC8403475; doi:10.7717/peerj.11936)
Supplement: Supplemental Information 9 — Mean reads per sample/MOTU from sequenced swipes of floor using cotton swabs, and sequenced faeces collected from the same floor. Non-meal MOTU are MOTUs that represent taxon that does not match plant items that were offered to the animals in the meal mixtures. Note that these data are from a pilot study, and not from the same faeces samples as in the main study. The process for data extraction and bioinformatics was the same as in the main study. [file peerj-09-11936-s009.docx]

**Supplemental Table S5.** Mean reads per sample/MOTU from sequenced swipes of floor using cotton swabs, and sequenced faeces collected from the same floor. Non-meal MOTU are MOTUs that represent taxon that does not match plant items that were offered to the animals in the meal mixtures. Note that these data are from a pilot study, and not from the same faeces samples as in the main study. The process for data extraction and bioinformatics was the same as in the main study.

|  | Swipe of floor in cage (n=30) | Faeces (n=30) |
| --- | --- | --- |
| Reads per sample | 38,928 ± 4,896 | 100,810 ± 10,402 |
| Reads per MOTU | 4,325 ± 523 | 22,911 ± 2,824 |
| Reads per non-meal MOTU | 1,708 ± 495 | 4,284 ± 1,895 |
